# Reviewer Acknowledgement 2013

**DOI:** 10.1186/1754-9493-8-5

**Published:** 2014-01-29

**Authors:** Philip F Stahel, Pierre-Alain Clavien

**Affiliations:** 1 Denver Health Medical Center, Denver, USA; 2 University Hospital Zurich, Zurich, Switerzland

## Abstract

**Contributing reviewers:**

The Editors of *Patient Safety in Surgery* would like to thank all our reviewers who have contributed to the journal in Volume 7 (2013).

## 

Mohamad Allaf

United States of America

Rodrigo Banegas

United States of America

Arne Berner

Germany

Shay Bess

United States of America

Jon Braverman

United States of America

Jonathan Bravman

United States of America

Evalina Burger

United States of America

Franco Cavaliere

Italy

Ted Clarke

United States of America

Christoph Eingartner

Germany

Mats Ekelund

Sweden

Federica Faggian

United Kingdom

Johannes Fakler

Germany

Joan Figueras

Spain

Michael Flierl

United States of America

Michael Frink

Germany

Beat Gloor

Switzerland

Dieter Hahnloser

Switzerland

David Hak

United States of America

Amy Halverson

United States of America

Eric Mark Hammerberg

United States of America

Kirk Hogan

United States of America

Kyros Ipaktchi

United States of America

Thilo John

Germany

Reto Kaderli

Switzerland

Enes Kanlic

United States of America

Jeffry Kashuk

United States of America

Stephen Kates

United States of America

Fernando Kim

United States of America

Senat Krasnici

Germany

Philipp Lenzlinger

Switzerland

Shalin Maheshwari

India

Lucas McCormack

Argentina

Timothy Moore

United States of America

John Mukhopadhaya

India

Matthias Münzberg

Germany

Frank Muscara

Australia

Giuseppe Nigri

Italy

Lena Nilsson

Sweden

Hans Oestern

Germany

Dipen Parekh

United States of America

Marco Penalonzo

Guatemala

Jean-Claude Roujeau

France

Ellen Sarcone

United States of America

Daniel Scheidegger

Switzerland

Hagen Schmal

Germany

Samuel Smith

United States of America

Wade Smith

United States of America

Martin Stockmann

Germany

Robert Stovall

United States of America

Chun-Hao Tsai

Taiwan

Todd VanderHeiden

United States of America

Michael Victoroff

United States of America

Charles Vincent

United Kingdom

Sebastian Weckbach

United States of America

Clement M.L. Werner

Switzerland

Kathryn Witzeman

United States of America

Heather Young

United States of America

George Youngson

United Kingdom

